# Determination of migration monomer styrene from GPPS (general purpose polystyrene) and HIPS (high impact polystyrene) cups to hot drinks

**DOI:** 10.1080/15376510802510299

**Published:** 2009-06-30

**Authors:** Mohammad-Reza Khaksar, Mahmoud Ghazi-Khansari

**Affiliations:** Department of Pharmacology, School of Medicine, Tehran University of Medical Sciences, Tehran, Iran

**Keywords:** Migration, Styrene monomer, Cups, Hot drinks

## Abstract

In this study, 162 samples were analysed for monomer styrene content with using high performance liquid chromatography (HPLC) method in hot tea, milk, cocoa milk. The monomer styrene content, expressed in μg/l of drink and the level of migration of styrene monomer were varied from 0.61 to 8.15 for hot tea, from 0.65 to 8.30 for hot milk, from 0.71 to 8.65 for hot cocoa milk in GPPS (general purpose polystyrene), from 0.48 to 6.85 for hot tea, from 0.61 to 7.65 for hot milk, from 0.72 to 7.78 for hot cocoa milk in HIPS (high performance polystyrene) cups in different temperatures and times. The estimated limit of detection of (HPLC) method for all samples was 0.001 mg/kg. There is linear regression for styrene monomer from 1 to 10 ng/ml. Several samples spiked with a known amount of styrene monomer. The results of the recovery in study for styrene monomer were determinate to be mean, 96.1 ± 1.92 to 99.7 ± 1.15%. The results of this study indicate that styrene monomer from polystyrene disposable into hot and fat drinks was migrated and this migration was highly dependent on fat content and temperature of drinks. The derived concentration of styrene monomer in this study was above the EPA (Environmental protection agency) recommended level, especially in MCLG (Maximum contaminant level goal) standard. More study is needed to further elucidate this finding.

## Introduction

Migration of substances from packaging materials into foods have been a major concern in recent years worldwide. There has been widespread concern on adverse effects of food products and food safety (Arvanitoyannis and Bosena 2004). Styrene monomer is one of the most widely used food packaged contact polymers. Increases in temperatures used in polymer processing may cause degradation and migration of polymers in food products (Till et al. 1982). Residual monomer levels in food contact plastics can be made less with good manufacturing processes, but it is not irrational to expect polystyrene food packaging materials to contain some styrene monomer and to expect the migration of some of it into foods and drinks (Till et al. 1987). Styrene was discovered in 1831. However, it did not become commercially important until 1942 when it was used in the synthesis of unsaturated polyesters and reinforced plastics (Tossavainen 1978; Windholz and Budavari 1983).

A significant amount of the polystyrene plastic material is used in many foods–contact applications (Lickly et al. 1995).

The main food contact applications of polystyrene are in dairy products including yogurt, cream, cottage cheese, ice cream and fruit juice, meat trays, biscuit trays, egg cartons, and drink cups (Modern Plastic 1991).

In recent decades, styrene monomer has been markedly used in manufacturing of disposable drinking containers; however, the extent of migration of residue styrene from polystyrene cups under different conditions is interesting in order to predict potential exposure of consumers to styrene from food–contact polymers (Tawfic and Huyghebaert 1998). Packaging and disposable service wares have mostly used styrene plastics (Watson and Wallace 1985). The migration of residual styrene from ‘solid’ polystyrene into food and food simulants has been extensively studied previously (Reid et al. 1980; Till et al. 1982, 1987).

Several adverse health effects are attributed to styrene. Human exposure to vapors of styrene may cause irritations of eye, nose, throat, and skin. Also, styrene has shown a toxic effect on the liver, and acts as a depressant on the central nervous system and cause neurological impairment (Varner and Breder 1981; Varner et al. 1983; Muratak et al. 1991; Cohen et al. 2002).

Chronic effects of styrene monomer and styrene epoxide (metabolite of styrene) are chromosomal aberrations in lymphocytes of humans and damage to the liver and nervous system. In recent years, studies of the toxic effect of styrene have given widespread concern on the haematopoietic, central nervous and peripheral nervous, ingestion, reproductive organs, and lymphatic system (Emma et al. 2001).

There is limited suggestion that styrene can cause any specific developmental or reproductive or endocrine toxicity. Other studies indicated that styrene may have effects on neurobehavioral development and neurotoxic actions (Lehr et al. 1993; Brown et al. 2000).

Styrene is metabolized via a number of different pathways. The most important metabolic pathway in humans is the conversion of styrene to styrene epoxide by the cytochromP450-mediated mono-oxygenize system in liver microsomes (Jenkins and Fennell 1994).

Biotransformation of styrene monomer to styrene epoxide and formation of peroxide radical is a dangerous problem for human health. For wider information on the pharmacokinetics; metabolism and general toxicity of styrene oxide, some reviews are available (Parrki 1985). Much of the toxicity of styrene monomer has been attributed to styrene-7, 8-oxide or styrene epoxide, whereas there are few data on the occurrence of styrene epoxide in packaging materials (Bond 1989).

Belvedere et al. (1984) have shown that isolated rat hepatocytes acted more efficiently in converting styrene oxide, suggesting that hepatocytes could be one of the most suitable systems for the study of indirect mutagens in vitro.

Residual monomer in food–contact polymers determinate in compare to EPA (environmental protection agency) standards which include MCL (Maximum Contaminant level) and MCLG (Maximum contaminate Level goal) (EPA 1995).

High performance liquid chromatography (HPLC) has become extremely popular and noticeable in recent years, and has been used to determine styrene in food products and in polystyrene packaging materials (Gawell and Larsson 1980; Varner and Breder 1981). Styrene monomer migration to the food products was analysed by GC-FID (Jin et al. 2005).

In this study, reversed phase HPLC was used to obtain styrene monomer level in some hot drinks that are readily available in markets from Tehran. The work in this paper determines the level of food contamination by styrene monomer derived from food contact materials, especially disposable drinking containers (GPPS, HIPS), and studies the effect of different parameters including the fat content, temperature, and time on the migration level, and compares the ability of different food stuffs to induce the migration of styrene monomer. It is expected that the results of this study will assist in acquiring information about the level of value of excess to limit this monomer.

## Materials and methods

### Apparatus

All glassware was soaked overnight in 15% (v/v) nitric acid, followed by washing with 15% (v/v) hydrochloric acid, and rinsed with double distilled water and dried before using.

The Hewlett Packard and waters associates' liquid chromatography systems were used for analysis of samples. Apparatus consisted of waters associates HPLC with a WISP automatic sample injector, 6000A solvent delivery system, with u.v. absorbance detector and a data module integrator/recorder. Chromatographic analysis was performed with an HPLC (Model water 600 controllers). The column (250.4.6 mm; id: 5μm) was packed with 6 μm Zorbax (ODS1), and the mobile phase consisted of 1.0 l of an aqueous solution of Water (25%)–Acetonitrile (75%). The flow rate was 1.0 ml/min and the sample injection volume was 50 μl. Ultraviolet detection (u.v.) was at 245 nm and AUFS of the system was 0.005.

A Hewlett Packard HPLC model (1084B) with model (RP-18) terminal and equipped with variable volume injector and variable wavelength detector was used. The mobile phase was distilled water (40%)–acetonitrile (60%) at a flow rate of 2 ml/min. Detection wavelength, column, and packaging and injection volume were as above. Attenuation was 0.075 AUFS.

### Reagents

All reagent used were of analytical reagent grade (Merck, Germany). Styrene (99%), Acetonitrile (HPLC grade with purity > 99%), Calcium chloride (20%), and redistilled water were obtained from across the chemical laboratory. Standard stock solution of styrene monomer was prepared from styrene (99%) added to methanol (HPLC-grade) as 90.6 mg/l and from this solution was used directly for experiments of calculation of standard curve and recovery styrene.

### Sample preparation and analysis

Different samples involved in this study included: Hot drinks (tea, milk, cocoa milk) obtained as dry powders, and reconstituted with boiling distilled water before use. Food stimulants: 3% acetic acid, 15% ethanol, and olive oil. Clear polystyrene cups or GPPS cups and mat polystyrene cups or HIPS cups (volume 200 ml, surface area 1.2 dm^2^), which are commonly used for hot drinks.

Samples were purchased from markets and stored at 4°C until analyzed. The samples were usually stored for no longer thon 7 days before being analyzed. The preparations of samples were prepared by the same procedure used by Flanjak and Sharrard (1984). The chromatography was carried out and peak heights were plotted against styrene concentration. By extrapolation, the amount of styrene in the sample was obtained. Calculation of the styrene concentration was by HPLC software based on peak area of the chromatograms of styrene obtained from the sample and calibration standard and the peak areas of the internal standards.

The calculation of recovered styrene was done according to the Calibration curve method by addition (2.5, 5, 10, 30, 50 mg/l) of styrene working solution (90.6 mg/l in methanol) to a styrene-free sample of the drinks (calibration standard). An identical volume of internal standard was added to the distillate and the sample. The blank and calibration standard solutions were also analyzed in the same way as the samples were prepared. The retention times for styrene and α-methyl styrene were approximately 4.6 and 6.5 minutes, respectively. In the food stimulants (acetic acid, ethanol, olive oil), styrene was distilled and determined by the same procedure. A known amount of styrene monomer (0.01 mg/kg) was added to some drink samples to determine recovery of styrene monomer from different drinks. The limit of detection of the analytical method for styrene analysis in drink samples was 0.001 mg/kg.

## Results and discussion

One hundred and sixty-two samples, including crystal and impact cups with hot different drinks such as tea, milk, and cocoa milk, were analyzed for determination of migration of styrene monomer. In all cases migration occurred and the amount of styrene monomer leached was in the range of 0.0012 mg/dm^2^ for crystal and impact cups with tea, milk, and cocoa milk. Our finding of styrene detection with HPLC method is similar to the study of Flanjak and Sharrad (1984) when they used both GC-FID and HPLC methods. However, our limit of detection was lower (0.001 mg/kg) than the HPLC (0.005 mg/kg) and GC-FID (0.01 mg/kg) methods of Flanjak and Sharrad (1984) when styrene monomer was determined in the food packaging of dairy products. This may indicate that our HPLC method of extraction and detection is more sensitive than the GC-FID and HPLC methods of Flanjak and Sharrad (1984). The concentrations of styrene monomer from these samples are presented in Tables 1 to 6 with means and SD. The results indicate that concentration varied from 0.61 to 8.15 μg/l for crystal cups with hot tea, from 0.65 to 8.30 μg/l for crystal cups with hot milk, from 0.71 to 8.65 μg/l for crystal cups with hot cocoa milk, from 0.48 to 6.85 μg/l for impact cups with hot tea, from 0.61 to 7.65 μg/l for impact cups with hot milk, and from 0.72 to 7.78 μg/l for impact cups with hot cocoa milk with different temperatures and times of storage.

**Table 1:** Contents of migration of styrene monomer from GPPS cups (μg/l) into hot tea in different temperatures and time periods.

	Time (min)		
	
Temperature (°C)	10	30	60
20	0.0	0.61 ± 0.01	1.24 ± 0.02
60	3.6 ± 0.04	3.95 ± 0.01	4.3 ± 0.06
100	7.50 ± 0.06	7.85 ± 0.04	8.15 ± 0.02

Data are means of three samples of three replicates.

**Table 2:** Contents of migration of styrene monomer from GPPS cups (μg/l) into hot milk in different temperatures and time periods.

	Time (min)		
	
Temperature (°C)	10	30	60
20	0.0	0.65 ± 0.01	1.02 ± 0.01
60	3.70 ± 0.03	3.99 ± 0.02	4.42 ± 0.03
100	7.82 ± 0.08	8.02 ± 0.04	8.30 ± 0.03

Data are means of three samples of three replicates.

**Table 3:** Contents of migration of styrene monomer from GPPS cups (μg/l) into hot cocoa milk in different temperatures and time periods.

	Time (min)		
	
Temperature (°C)	10	30	60
20	0.0	0.71 ± 0.04	1.24 ± 0.02
60	3.72 ± 0.03	4.02 ± 0.09	4.53 ± 0.02
100	8.02 ± 0.05	8.28 ± 0.01	8.65 ± 0.01

Data are means of three samples of three replicates.

**Table 4:** Contents of migration of styrene monomer from HIPS cups (μg/l) into hot tea in different temperatures and time periods.

	Time (min)		
	
Temperature (°C)	10	30	60
20	0.0	0.48 ± 0.02	1.02 ± 0.03
60	3.24 ± 0.05	3.22 ± 0.09	3.88 ± 0.06
100	6.04 ± 0.07	6.25 ± 0.02	6.85 ± 0.04

Data are means of three samples of three replicates.

**Table 5:** Contents of migration of styrene monomer from HIPS cups (μg/l) into hot milk in different temperatures and time periods.

	Time (min)		
	
Temperature (°C)	10	30	60
20	0.0	0.61 ± 0.01	1.27 ± 0.03
60	3.82 ± 0.02	4.15 ± 0.01	3.98 ± 0.01
100	6.94 ± 0.03	7.12 ± 0.03	7.65 ± 0.02

Data are means of three samples of three replicates.

**Table 6:** Contents of migration of styrene monomer from HIPS cups (μg/l) into hot cocoa milk in different temperatures and time periods.

	Time (min)		
	
Temperature (°C)	10	30	60
20	0.0	0.72 ± 0.02	1.34 ± 0.07
60	3.99 ± 0.02	4.32 ± 0.09	4.13 ± 0.04
100	7.02 ± 0.03	7.28 ± 0.03	7.78 ± 0.03

Data are means of three samples of three replicates.

Statistical analysis of results by ANOVA showed no significant differences among all samples. Recovery of added styrene monomer from various drinks is presented in Table 7. The range of recovery was 96.1 to 99.7%, with the lowest recovery belonging to Cocoa milk, and the highest recovery belonging to tea.

**Table 7:** Recovery of added styrene monomer in various drink samples.

Type of drink	Monomer styrene added(mg/l)	Monomer styrene(mg/l)	% Recovery
Tea	0.01	0.0997	99.7
Milk	0.01	0.0972	97.2
Cocoa milk	0.01	0.0961	96.1

Data are means of three determinations from three separate samples.

Exposure conditions were chosen based on a number of criteria. In the case of simulants, the European Community test conditions (EEC 1985) close to those of exposed use were chosen (24 h at 40°C and 1 h at 100°C), and these conditions are mentioned in Directive93/8/EEC also (EEC 1993).

Data from reproducibility of studies demonstrates adequate reproducibility for routine laboratory use. Migrations of styrene monomer into aqueous drinks were lower than of the milk and cocoa milk under the same test condition. Varner and Breder (1981) reported that hot coffee and hot water extract styrene monomer in the same extent.

Directive 93/8/EEC lays down the test condition to be chosen according to condition of contact in actual use. In our migration experiments, we applied different conditions, as our main interest was focused on the actual use of these cups and the comparison between the different storage times and temperatures (EEC 1993).

Determination of migration level in beverage products can cause analytical difficulties due to complex composition of most foodstuffs. Consequently it is necessary to use both conventional food simulants and standardized test conditions, which are meant to simulate the migration of substances into foods under valid conditions of use (O'Neill et al. 1994; Synder and Breder 1985). Directive 82/711/EEC lists the following food stimulants: (a) distilled water of equivalent quality; (b) 3% (w/v) acetic acid in aqueous solution; (c) 15% (v/v) ethanol in aqueous solution; and (d) rectified olive oil. In the current study acetic acid (3%), ethanol (15%), and olive oil were used as food simulants. The results obtained from migration tests in this study are summarized in Table 8, and are in agreement with the results of Tawfic and Huyghebaert (1998) and Ramshow (1984), reported that the amount of migration styrene of monomer is higher in products with higher fat content.

**Table 8:** Migration of styrene monomer from polystyrene cups into drink simulants (mg/l).

		Styrene residues in	
		
Simulants	Test condition	GPPS cups	HIPS cup
acetic acid	1 h at 100°C	0.074	0.054
	24 h at 40°C	0.088	0.068
15% Ethanol	1 h at 100°C	0.052	0.041
	24 h at 40°C	0.067	0.051
Olive oil	1 h at 100°C	0.028	0.025
	24 h at 40°C	0.034	0.029

Results presented as an average of three determinations from three separate samples.

Flanjak and Sharrad (1984) determined the migration of styrene monomer from polystyrene cups into different samples including fat products, suggesting that because of the hydrophobic nature of styrene, the migration of this monomer into fat beverages ise higher than in aqueous drinks.

**Figure 1:**
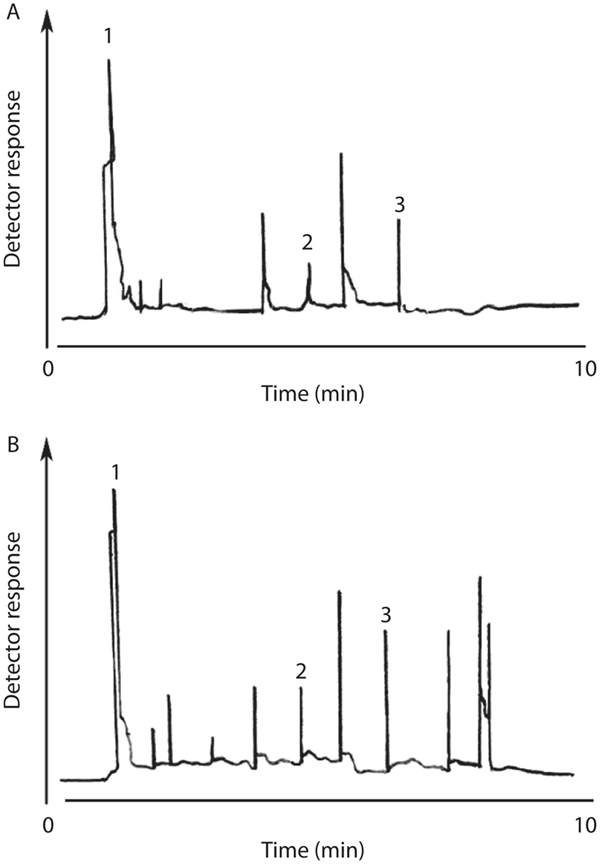
HPLC chromatogram for migration of styrene monomer from hot milk to GPPS (A) and HIPS (B) cups. Mobile phase is acetonitrile (1) and internal standard is—methyl styrene (3). (a) Styrene corresponds to 8.30 ng/l of styrene (2) with a peak height of 12% at an attenuation setting of 0.075 AUFS. (b) Styrene corresponds to 7.65 ng/l of styrene (2) with a peak height of 11% at an attenuation setting of 0.075 AUFS.

The results of this study indicated that migration of styrene monomer from polystyrene cups into hot beverages when temperature increased is considerable (Tables 1 to 6). The retention times for styrene monomer and α-methyl styrene were approximately 4.5 and 6.5 minutes, respectively. Typical hot milk chromatograms are shown in Figure 1. In both chromatograms, the mobile phase was acetonitrile and attenuation was 0.075.

## Conclusion

The results in this study indicated that the migration of styrene monomer from polystyrene cups into different beverages does not increase by more than 0.05% of the total amount of styrene in the cup. The migration of styrene into hot beverages essentially depended upon the fat content, storage temperature, and time. However, temperature has an important role in leaching of styrene monomer from polystyrene cups, as mentioned above. Migration testing employing aqueous food simulants will necessarily lead to under-estimation of the migration processes occurring in real food products. However, the amount of styrene monomer migration at 20°C is minimal, but when temperature increased, the migration in the first 10 minutes is considerable, and increases as the time of exposure increases. Furthermore, the level of migration of styrene monomer into hot cocoa milk was more than hot milk and the migrations of styrene into hot milk was more than tea with the same conditions and essentially affected by the time of contact.
